# Quinine Rash

**Published:** 1879-09

**Authors:** R. J. Nunn

**Affiliations:** Savannah, Ga.


Medical and Surgical Journal.

Vol. XVII.] SEPTEMBER—1879. [No. 6.

### Original Communications.

### QUININE RASH.

By R. J. Nunn, M.D., Savannah, Ga.

During a discussion upon the "Rash" following the administration of quinine, which took place a year or so ago at a meeting of one of the medical societies in London, England, attention was drawn to the meagreness of medical literature on this subject. It is with the intention of assisting to remedy this want that the following cases are placed upon record.

A lady about fifty years of age, of a nervous, sanguine and highly phlegmatic temperament, was in 1854 attacked with yellow fever, dating from which time the production of quininism in her was always accompanied with the appearance of bright red, dry erythematous patches in five places, viz.: the left cheek and all of the nose, the chin, rather towards the left side, the palmer aspect of the left wrist, the back of the left hand over the carpo-phalangeal articulations of the fourth and fifth fingers, and the left knee.

The spots were from an inch to an inch and a half in diameter. The line of demarkation between the red spot and the unchanged skin was clear and well marked. There was no shading off, no blush, nor any change of color of the surrounding cuticle. There was a sensation of burning and itching in the patches and after a few days the spots would desquamate and disappear to reappear whenever the system was again saturated with quinine.

The quantity of quinine which served to produce this eruption varied very considerably, sometimes five grains would call it forth, and again thirty grains might be required to cause it to appear, but, always it was the precursor of quininism and could be depended on as a certain sign that the medicine had produced its full effect upon the system.

No other medicine which has been administered to this patient has ever caused a like effect, nor did indigestion, constipation or any other affection or accident of the system produce it ; but now comes the most unusual part of the case.

In the spring of the year (1879) this patient had an attack of pneumonia, during which and since that time, quinine has ceased to produce the erythematous eruption described or to give rise to any symptoms other than the ordinary therapeutic effects.

A case reported to the Georgia Medical Society by Dr. J. C. Habersham is that of a female patient in whom the administration of quinine always produces so extensive and disagreeable an eruption on the palms of the hands and on the face that the medicine has to be discontinued. Arsenic and other remedies did not cause any similar results.

Dr. J. B. Read permits me to mention a case in which quinine produced desquamation of the whole body—as after a severe attack of scarlet fever—to such an extent that he was enabled on two occasions as the result of the administration of quinine to obtain and lay before the Georgia Medical Society perfect and complete cuticular casts of the hand and feet of this patient.

